# Family Violence Risk on Entry to the Family Courts of Australia: Profiles and Predictive Validity of the DOORS Triage Process

**DOI:** 10.1177/08862605251363611

**Published:** 2025-08-22

**Authors:** Yvonne D. Wells, Jennifer E. McIntosh, Felicity L. Painter, Amy Holtzworth-Munroe, Bradley J. Wright, Heng Jiang, Jamie Lee, Kristel A. Krella, Anna T. Booth, Elizabeth Evans

**Affiliations:** 1 La Trobe University, Melbourne, VIC, Australia; 2 La Trobe University, The Bouverie Centre, Melbourne, VIC, Australia; 3 Indiana University, Bloomington, USA; 4 Relationships Australia South Australia, Adelaide, Australia; 5 Federal Circuit and Family Court of Australia, Melbourne, VIC, Australia

**Keywords:** triage, risk, screening, family violence, separation, court

## Abstract

The Family DOORS Triage Tool is a self-report measure of family violence victimization and perpetration risk, completed by parties on filing or responding to a parenting matter in the Federal Circuit and Family Court of Australia (Divisions 1 and 2), collectively “the Courts.” The tool and associated risk rubric inform subsequent risk response processes within the Courts. We sought to test the tool’s utility and fitness-for-purpose within a new family violence response initiative by the Courts. Data from 4,175 adults, collected using the Family DOORS Triage Tool and Court intake records, were used to examine (a) self-reported family violence risk profiles and risk factor clustering; (b) the tool’s acceptability across key sub-groups (using time to completion as a measure of engagement); and (c) the tool’s fitness-for-purpose against the results of subsequent file review by designated court officers. Concern for one’s own safety was the most frequently reported risk. Allocation to a high-risk category was associated with gender (higher for women), applicant status (higher for respondents), culturally and linguistically diverse background status, more recent separation, recent communication with the other party, and having younger children. Survey completion was fastest for those who reported high risk or identified as Aboriginal and/or Torres Strait Islander. Risk category was a strong predictor of subsequent referral to the high-risk case management pathway following assessment and file review. Parties who initially self-reported high risk but were reclassified to low/medium risk on file review were more likely to be men, to report low fear for their own safety, and to be in recent communication with their former partner. The Family DOORS Triage Tool coupled with the Court’s risk rubric demonstrates high utility and acceptability to vulnerable sub-populations. The tool may provide a means to detect and respond rapidly and accurately to clusters of risk early in engagement with family court systems.

## Confidential Family Safety and Risk Screening

The dual processes of parental separation and turning to a family law court to resolve parenting issues combine to create escalating risks to well-being and safety, especially for women and children (Campbell et al., 2003; Spearman et al., 2023). Stalking, harassment, psychological, verbal, and emotional abuse that emerge around the time of separation increase significantly with the commencement of litigation over parenting arrangements (Romero & Staudenraus, 2024; Tomlinson et al., 2021).

In the Australian context, arguments have mounted for the family law courts to embrace a public health role, by ensuring early, confidential family violence risk screening and triage processes. For example, the Australian House of Representatives Standing Committee on Social Policy and Legal Affairs (2017) called on the family law system to screen for risk with system-specific tools (pp. 84–88, Rec 2) and identified barriers to the effectiveness of risk screening, including the admissibility of family violence evidence. Women were found to under-report risks to their safety, due to fear of recrimination that may flow from admissible affidavit material.

### A Brief Precis of Australian Family Law Process and Procedures

We note that procedures and terminology in the Australian Family Law system largely align with U.K./Commonwealth Family Law systems but differ from the U.S. legal system in important ways. In Australia, the Commonwealth (federal government) has jurisdiction over marriage, divorce, and parenting and property disputes on separation for both married and de facto couples, while states and territories handle child protection and domestic and family violence protection orders (DFV; noting new DFV initiatives below in the Courts).

All U.S. states now recognize no-fault divorce. As of 2023, 17 states and the District of Columbia provide only for no-fault divorce, with the remaining states also providing for tailored consideration of fault in specific instances, such as abandonment and cruelty (Butler, 2025; Felton & Schweiger, 2025). In Australia, the only requirement for a divorce application is that the marriage has broken down irretrievably. Couples separated for at least 12 months (even if living under the same roof), with no reasonable likelihood of resuming the marriage, can make an application for a divorce (*Family Law Act 1975* (Cth) pt VI ss 48, 49). This can be done either jointly (both spouses make the application together) or by one party, “the applicant,” to which the other party becomes “the respondent.” If the court is satisfied that the legal requirements have been met, a *Divorce Order* becomes final one month and one day after it is made (*Family Law Act* s 55). For context, beyond obtaining this order, the majority of separating families have no further engagement with the family law system (Kaspiew, 2022).

Disputed matters (property and/or parenting) are dealt with separately, and additional legal steps are required. Before filing for parenting orders (custody orders in the United States), parents are required to make “a genuine effort” to resolve the matter through Family Dispute Resolution (FDR), a form of mediation (*Family Law Act* s 60I). In some circumstances, particularly in cases involving family violence, exemptions are made, and FDR is not required. A Consent Order can be issued when parents choose to make legally binding their mediated/agreed decisions regarding parenting, property, and/or financial matters.

Those families unable to resolve their issues privately or with community-based FDR will use lawyer-led negotiation and/or the Courts to assist. These families are more likely to report domestic and family violence risk (Kaspiew, 2022). For contested parenting matters (the focus of the current study), *Parenting Orders* are made that set out the arrangements regarding children. This can include Parental Responsibility (decisions about the child’s upbringing, e.g., education, healthcare, religion); Live with/Spend Time with (where the child will live and how much time they will spend with each parent); Communication (how the child will communicate with a parent they do not live with or other people); and any other aspect of the care, welfare, or development of the children (schooling, medical treatment, and other relevant matters). *Financial and Property Orders* (including spousal maintenance) can be made alongside Parenting Orders. In considering orders made about children, the Court must consider what is in the child’s best interest (*Family Law Act* s 60CC), including arrangements to promote safety and mitigate the risk of family violence (*Family Law Act* s 60CG).

### The Lighthouse Project: A New Family Violence Initiative of the Courts

In 2019, new funding was awarded to the Federal Family Law Courts to support families affected by family violence. This funding enabled piloting of the Court-initiated risk screening and triage program for matters being considered by family law courts, with three interconnected processes applied to parenting matters: self-report screening for family safety risks at the point of filing; confirming high-risk status through interview and file review in order to triage matters to an appropriate pathway; and maintaining a specialist list, which came to be known as the Evatt List, to hear matters assessed as involving a high risk of family violence and other family safety risks. To support the pilot and its objectives, the Courts sought an amendment to the *Family Law Act 1975* (Cth), the *Family Law Amendment (Risk Screening Protections) Act 2020*, allowing for the family safety risk screening process to be confidential and inadmissible as evidence.

The collective risk screening, triage process, and specialist list were initially piloted in three family law registries (Adelaide, Brisbane, and Parramatta for parenting/custody-only matters from December 7, 2020 to November 27, 2022), and was known as the Lighthouse Project Pilot, as one of several initiatives from the Courts to address high levels of family violence affecting parties and their families (given 60% of parties are designated high risk: Federal Circuit and Family Court of Australia, 2023).

### The Family DOORS Triage Tool

The primary element of screening in the Lighthouse Project Pilot was the DOORS Triage Tool (McIntosh et al., 2020; McIntosh et al., 2023), referred to in the Courts as Family DOORS Triage. The tool draws on the structure of the original Family Law DOORS (FL-DOORS; McIntosh & Ralfs, 2012), used in community mediation and counseling services with separating parents. This is a 10-domain all-of-family risk and well-being screening measure. It has been validated twice against external safety criteria, with over 6,000 cases including 1,642 paired parties to a matter (McIntosh et al., 2016; Wells et al., 2018). Each study found robust psychometric properties and fitness-for-purpose as an indicator of family violence risks.

In 2020, the Courts commissioned an adaptation of this tool, for use as a self-report online screen at the front end of the triage process. The DOORS Triage Tool retained the original 10 domain structure of the FL-DOORS and a modified three-step risk appraisal process (DOOR 1 Self-report, DOOR 2 Elaboration on needs for support, and DOOR 3 Response plan). The Courts’ own internal risk classification rubric (co-developed with DOORS Triage Tool authors) was applied to responses, to determine overall high, medium, or low risk status. The method for completion of the tool and subsequent triage processes is described in the “Methods” section.

During its pilot phase, 73% of eligible parties completed the DOORS Triage Tool in the Adelaide, Brisbane, and Paramatta registries (Federal Circuit and Family Court of Australia, 2023). An independent evaluation commissioned by the Courts (Nous Group, 2022) demonstrated that the Pilot’s strategic intent, to improve the Courts’ identification and response to risk, was being realized and that the use of DOORS Triage as part of the Pilot was an efficient and reliable tool to identify risk at scale. Findings supported expansion of the model in the Courts and inclusion of both parenting-only, and parenting and financial, matters. The Courts subsequently expanded Lighthouse to 15 family law registries, following funding as part of the 2022 to 2023 Federal Budget from November 28, 2022 (Adelaide, Brisbane, Cairns, Canberra, Dandenong, Darwin, Hobart, Launceston, Melbourne, Newcastle, Parramatta, Rockhampton, Sydney, Townsville, and Wollongong registries; Federal Circuit and Family Court of Australia, 2023).

### Study Rationale and Aims

At this stage in the development of the Lighthouse project, it was timely to evaluate the utility of the DOORS Triage Tool. In particular, it was important to: describe the risk profiles identified by the DOORS Triage Tool and the family variables associated with high risk, which speak to the tool’s perceived validity within the literature on family violence; assess the engagement of parties in Court family matters with the DOORS Triage Tool, which speaks to the tool’s perceived value to those parties; and compare the results of the DOORS Triage Tool with subsequent file review by designated court officers, which speaks to the fitness-of-purpose of the DOORS for Court intake processes. We also wished to explore gender as a factor in risk assessed at entry to the Court system.

The present study had three aims:

1. To describe the immediate and proximal family violence risk factors reported by applicants (the party that applies to the Court to commence legal proceedings against another person or persons) and respondents (a party to court proceedings against whom relief is claimed) on entry to the Courts, clustering of risk factors, and predictors of high-risk classification.
2. To test the acceptability of the DOORS Triage Tool across a subset of the Courts’ population, through examination of engagement and completion by risk status and cultural groupings.
3. To test the accuracy of self-reports of high risk on the DOORS Triage against subsequent risk level as assessed by designated court officers, leading to referral to standard or high-risk case management pathways.

This study is the first in a suite of studies tracking the utility and impact of early screening on family safety up to one year after filing or responding to parenting proceedings in the Courts.

## Method

The STROBE (Strengthening the Reporting of Observational Studies in Epidemiology; Von Elm et al., 2007) cross-sectional reporting guidelines were used to construct this report.

### Participants

Data reported here are from a subsample of the Courts’ population who: (a) were seeking parenting/custody orders (with or without financial or property orders) filed in an eligible family law registry; and (b) voluntarily elected to complete the DOORS Triage Tool at entry to court and agreed to share their data for research purposes. The sample comprised 4,175 parties screened between September 1, 2021 and September 12, 2023: 1,345 were screened during the pilot, and the remaining 2,830 in the expansion period of Lighthouse. The total sample included 487 applicant and respondent pairs (i.e., parties in the same matter). For the remaining cases (*n* = 3,234; 78%), only one party completed the DOORS Triage Tool within the data collection period and agreed to share their data. Matters including multiple applicants were excluded from the current sample.

### Recruitment Process

Sampling processes were carried out on secondary data (i.e., de-identified records originally collected by the Courts as part of standard processes). During the Lighthouse pilot and expansion periods, parties filing or responding to an eligible family law matter (i.e., parenting only proceedings during the pilot; parenting only, and parenting and financial, proceedings during the expansion period) were asked by the Courts to complete the DOORS Triage Tool via a confidential link to a secure online platform, using an electronic device (i.e., computer, smart phone, tablet). Parties were encouraged to complete the triage tool at the earliest opportunity, at a time when they had the privacy to do so. While no timeframe was enforced, the access code for the risk screen expired after a set amount of time; it could, however, be reset on request. Parties could save their responses and return to the risk screen, enabling completion over several sittings. If completed in one sitting, the triage tool takes approximately 10 min for a party with little or no risk to report, and 15 to 20 min when subsequent DOOR 2 and DOOR 3 questions are triggered by an affirmative response in DOOR 1. If needed, assistance to complete the screen was provided by the Lighthouse Team.

Opt-in consent for DOORS Triage screening data to be shared for research purposes was offered following completion of the tool. Data for consenting parties were fully anonymized and added to the DOORS Triage databank held by the Courts for later transfer to researchers for analysis. No financial inducements to share data were offered. As determined by the consent process, data were available for all registries except Newcastle. Ethics approval was provided by the Federal Circuit and Family Court of Australia Research and Ethics Committee (approval number 06072022) and confirmed by the La Trobe University Human Research Ethics Committee (HEC21175).

### Participant Characteristics

Altogether, 4,175 eligible parties filing in the Courts between September 1, 2021 and September 12, 2023 agreed to complete the DOORS Triage Tool and to share their data with the research team. Of these, 59.9% of the sample were applicants, and 40.1% were respondents. Of the participant group, 211 (5.1%) identified as from Aboriginal or Torres Strait backgrounds. This proportion is similar to the proportion of Indigenous parties involved in final order applications (4.4%–6.9%; 2018–2019 and 2022–2023) and is higher than the national population proportion (3.2%; Australian Bureau of Statistics, 2021). A small proportion of parties (*n* = 233, 5.6%) self-identified as culturally and linguistically diverse (CALD), based on their country of birth and/or main language. The average age of participants was 37.8 years (median 38 years), and the mean time since separation was 7 years (median 2.5 years) (see Table 1).

**Table 1.** Sociodemographic Characteristics and Family Structure of Parties by Gender (% or Mean [*SD*]).

Characteristic	Valid %	Men (*n* = 1811)	Women (*n* = 2358)	χ^2^	*p*
Applicant status (applicant)	59.9	72.9	50.0	225.4	<.001
Identifies as CALD (yes)	5.6	5.1	6.0	1.5	.216
Identifies as indigenous (yes)	5.1	4.2	5.7	5.0	.026
Location (metropolitan area)	67.7	70.0	65.8	8.2	.004
Ever lived together (yes)	95.1	95.4	94.8	0.8	.382
Communicated in past 6 months (yes)	81.9	85.6	79.1	29.2	<.001
Children’s age profile				2.3	.314
Young children only	34.0	34.8	33.4		
Older children only	52.2	50.8	53.2		
Both younger and older children	13.8	14.4	13.4		
Spent time with younger children (yes)	89.4	81.1	96.1	116.9	<.001
Spent time with older children (yes)	88.2	81.3	93.5	97.1	<.001
	Mean (*SD*)	Mean (*SD*)	Mean (*SD*)	*t*	*p*
Age	37.8 (8.0)	39.4 (8.4)	36.6 (7.4)	11.1	<.001
Time since separation (years)	3.4 (3.1)	3.2 (3.0)	3.5 (3.1)	3.5	<.001

*Note*. CALD = culturally and linguistically diverse.

More women than men completed the DOORS Triage Tool and consented to its use for research purposes (57% vs. 43% of the study sample). Only six cases (0.14% of cases) were coded as Unspecified on Gender. These cases were omitted from the analyses by gender but included in all other analyses. Women were more likely to be respondents to the matter than men, who were more likely to be applicants. Women were significantly younger than men, more likely to identify as Indigenous, and more likely to live in non-metropolitan areas. They reported longer time since separation than men (consistent with a higher proportion being respondents) and were less likely to have communicated with the other party in the past 6 months. The age profiles of the children of male and female parties were similar, but women were more likely than men to report having spent time in the previous 6 months caring for both younger and older children (see Table 1).

### Measures

#### Pre-Screening Data

When administering the risk screen, data fields pertaining to party demographic and familial structure are filled in by either the party themselves or by a designated court officer based on the individual party’s Initiating Application or Response to Initiating Application (a mandated court form completed by all parties at entry to court). Intake data used in the present study included postcode, gender, age, children’s age profile, Aboriginal and/or Torres Strait Islander status, and CALD background status.

#### DOORS Triage Screening Tool

The DOORS Triage Tool (McIntosh et al., 2020) comprises 10 risk domains retained from the original FL-DOORS, each exploring risks associated with: (1) cultural and religious background, (2) feelings about the separation, (3) managing conflict, (4) how the party is coping, (5) how the other party appears to be coping, (6a) the baby/young children (if any), (6b) the school aged children (if any), (7) managing as a parent, (8) the children’s safety, (9a) the party’s safety, (9b) if the party is behaving safely, and (10) other stressors. Risk in each domain is appraised in a three-step process (DOORs 1, 2, and 3). Parties are first asked to identify the presence of risk (DOOR 1), with dichotomous response options (Yes/No or Agree/Disagree). If present, additional questions are then asked exploring the need for support with respect to that risk (DOOR 2), followed by an indication of the party’s own safety-related responses in relation to the risk (DOOR 3). Completion of the DOORS results in a “risk profile.”

#### Overall Risk Classification

The DOORS Triage Tool provides a first, automated risk flag indicating the level of risk self-reported across the domains and highlighting patterns of risk and priority for follow-up. The Court’s risk rubric then provides an overall risk classification of High, Medium, or Low. This rubric is weighted to reflect risk reported within the DOORS Triage safety domains (Domains 4, 8, 9a, and/or 9b—see descriptors above).

#### Two-Step Independent Verification of High-Risk Referral Process

On completion, the party’s responses on DOORS Triage are first submitted directly to the Courts’ Lighthouse Team, which comprises Lighthouse support officers and triage counselors, for review against the Courts’ risk rubric. Following the triage process, parties are provided with a copy of their responses, a safety and well-being plan, and a list of specialist services. Those parties with high-risk markers have further clinical review and risk assessment by a triage counselor, a court employee who has skills and experience in risk assessment and holds a relevant degree (such as in social work or counseling), and whose role is to elaborate on the party’s responses regarding family safety and well-being risks as flagged in the tool. This is completed by way of a Case File Review and may also include a telephone interview, known as a Triage Interview, in which the Triage Counselor also provides personalized, tailored advice and referral suggestions.

Part IIA of the *Family Law Act 1975* (Cth) protects the confidentiality of the risk screen and prevents the disclosure and admission into evidence of information provided in connection with family safety risk screening. Therefore, the DOORS Triage Tool data and subsequent Triage Interview or risk classification status are confidential (except in cases of serious imminent risk of harm or harming or serious child protection concerns). This information does not form part of the court file but is used by the Triage Counselor to recommend an appropriate case management pathway.

Following overall risk classification against the Courts’ risk rubric by the Lighthouse Team, medium or lower risk parties are offered safety planning and service referrals as part of the secure online process, noting these matters are not reviewed by a triage counselor except in limited circumstances. The matters will be considered for a range of case management options, including FDR, and other specialized lists such as the Specialist Indigenous List, and directed to a suitable pathway based on the individual needs of the case. This is commonly referred to as the standard case management pathway. Where a party is at high risk, following review by a triage counselor, the matter may be referred to a judicial registrar to be considered for placement on a specialist court list, called the Evatt List. This list was developed to assist families identified as being at high risk of family violence and other safety concerns, through early information gathering and targeted intervention at the commencement of proceedings. The team, including judges, senior judicial registrars, and judicial registrars, has specialized training and is experienced in working with families where high-risk safety issues have been identified. Not all matters referred to the Evatt List will be placed on the List. This end-to-end Lighthouse screening, triage, and specialized case pathway process is multi-disciplinary and rests on extensive training of judges, registrars, court child experts, and staff, to ensure best practice implementation of these procedures and processes in identifying and responding to risk.

#### Calculated Variables

(a) The Modified Monash Model class (a measure of location remoteness and population size) was calculated from postcode and then reclassified as metropolitan or regional/remote as an indicator of access to services. (b) Submission time from click to completion (“Submission time”) was recorded as the time between first opening the link to the DOORS Triage Tool and final submission (i.e., finish time—time survey link was first opened). Submission time was then dichotomized (<72 min vs. 73 min or longer: see below) for analysis.

### Analytic Strategy

#### Analysis of Risk Factors

Frequencies, the χ^2^ statistic, and *t*-tests were used to describe the sample and parties’ risk profile by gender, applicant/respondent group, and low/medium versus high-risk category (following application of the Courts’ rubric to the DOORS triage risk profile). IBM SPSS Version 29.0 for Windows was used for these analyses.

Tetrachoric correlation is a technique used for calculating the relationship between two dichotomous variables that represent underlying continuous variables and permits factor analysis of dichotomous data. Stata Version 18.0 (StataCorp LLC) was used (a) to calculate tetrachoric correlations between dichotomized risk status (medium/low vs. high) on domains of the DOORS Triage Tool and (b) for factor analysis of the resulting tetrachoric correlation matrix.

The most proximal predictors of risk to safety as assessed across domains of the DOORS Triage Tool were identified in multiple logistic regression analyses (high vs. low or medium risk). Variables with bivariate *p* < .25 were entered into the regression equation, consistent with common practice (Hosmer et al., 2013), and backwards deletion was used to remove variables one at a time until a parsimonious model was achieved.

#### Engagement and Acceptability

The lag between opening of the link and completion of the DOORS Triage Tool is of interest. Given the voluntary nature of the screening process, it provides a proxy for engagement with and acceptability of the survey. Recorded times between opening the link to the survey and submitting it (“submission times”) were too highly skewed to use in parametric analyses. Frequencies on submission time increased up to 71 to 72 min and declined sharply after that. Therefore, submission times were described as short (72 min or less) or long (over 72 min). Submission time was explored first in bivariate analyses using the χ^2^ statistic and then in multivariate logistic regression analyses controlling for potential confounders.

#### Subsequent Confirmation

To examine the accuracy of the DOORS Triage self-reported risk classification (i.e., the Courts’ risk rubric applied to DOORS data) against later referral recommendation (risk pathway, as determined by Case File Review, and, where identified as potentially high risk, Triage Interview by a Triage Counselor), a direct comparison of the two indicators of risk was first undertaken. Then a series of bivariate analyses were undertaken to determine potential predictors of “false positives” (i.e., those allocated to the high-risk group by the DOORS Triage Tool but subsequently referred to a non-Evatt pathway vs. those who remained classified as high risk after assessment by a Triage Counselor). This analysis was followed by a multivariate logistic regression analysis to identify the most proximate predictors of pathway reallocation.

#### Treatment of Missing Values

The DOORS Triage Tool asks only about relevant risks, meaning some domains were applicable to only some parties. For example, parties who had not communicated with the other party in the previous 6 months were not asked about the other party’s capacity to parent, and risk to younger and older children was asked only if the family included children in that age group. There was an extremely low number of missing values for domain scores (maximum *n* = 3). Where necessary in multivariate analyses, participants who were not required to respond to a particular risk domain (i.e., not applicable) were coded as having zero risk in that domain. No other adjustment was made to account for missing values.

## Results

### Aim 1. Immediate and Proximal Family Violence Risk Factors Reported by Applicants and Respondents on Entry to the Courts, and Clustering of Risk Factors

This series of analyses was undertaken to describe self-reported family violence risk profiles, examine surrounding cultural and social-ecological factors associated with safety risk status, and assess the utility of the multi-domain tool.

#### Risk Profiles by Gender

The most common risk reported was concern for one’s own safety because of the behavior of the former partner, reported by 58.7% of the sample (see Table 2). Relatively high proportions of parties also reported concerns on the DOORS triage domains addressing their former partner’s mental health (Domain 4; 14.3%) and concerns for their children’s safety in the care of their other parent (Domain 8; 11.4%). The domain-specific prevalence of high risk on all other domains was <10%.

**Table 2.** Risk Profile of Parties by Gender (% High Risk).

Variable	High Risk on This Domain (Valid %)	Men	Women	χ^2^	*p*
Domain 1 Fear for cultural/religious context	8.2	5.4	10.5	35.5	<.001
Domain 2 Negative emotions	0.6	0.9	0.3	6.2	.013
Domain 3 Serious current conflict	1.1	1.4	0.9	1.9	.164
Domain 4 Own mental health and coping	6.9	6.6	7.2	0.5	.493
Domain 5 Other party’s mental health and coping	14.3	16.1	12.7	8.3	.004
Domain 6a Young children’s distress	2.9	5.3	1.2	25.2	<.001
Domain 6b Older children’s distress	3.5	5.9	1.8	29.2	<.001
Domain 7 Parenting difficulty	1.7	3.1	0.8	28.5	<.001
Domain 8 Concerns for children’s safety	11.4	13.2	10.1	8.2	.004
Domain 9a Concerns for own safety	58.7	44.4	69.7	269.7	<.001
Domain 9b Safety of own behavior	4.3	6.2	2.8	28.7	<.001
Domain 10 Life stresses	4.1	6.0	2.5	31.8	<.001

Women were much more likely than men to report being afraid for their safety (Domain 9a) on the DOORS Triage Tool and were nearly twice as likely to report risk due to cultural or religious factors (Domain 1). In contrast, men were more likely to report risk associated with their own negative feelings about separation (Domain 2), concern about the other party’s mental health (Domain 5), perceived distress in both young and older children (Domain 6a and 6b), difficulty parenting (Domain 7), concerns for their children’s safety (Domain 8), concerns about their own behavior (Domain 9b), and life stresses (Domain 10).

#### Applicant/Respondent Status

Systematic differences between applicants and respondents were identified (see Supplemental Table 2 for details). Domain-specific risk on the DOORS Triage Tool was generally similar for applicants and respondents. However, respondents were significantly more likely than applicants to report concerns for their own safety because of the behavior of their former partner (Domain 9b). Applicants were more likely than respondents to report distress in both younger and older children (Domain 6a and 6b), difficulty parenting (Domain 7), and concerns about their own behavior (Domain 9a; see Supplemental Table 3).

#### Associations With and Within Low/Medium Versus High-Risk Self-Reports

Almost two-thirds of respondents (61.8%) were classified as being in the high safety risk group following application of the Courts’ overall risk rubric to DOORS triage data. Several sociodemographic and family characteristics were associated with overall high-risk category on DOORS domains (see Table 3). In a multiple logistic regression with backward deletion of variables, the most important independent predictors of allocation to a high-risk category were respondent status, being a woman, CALD background status, not having communicated with the other party in the previous 6 months, and shorter time since separation. The odds for women being classified as high risk were over two-and-a-half times the odds for men, other factors being equal (see Supplemental Table 4).

**Table 3.** Parties’ Profiles by High or Low/Medium Risk Status on the DOORS Triage Tool (% or Mean [*SD*]).

Characteristic	Low/Medium Risk (*n* = 1,594)	High Risk (*n* = 2,581)	χ^2^	*p*
Applicant status	67.4	55.3	59.6	<.001
Gender (male)	58.3	34.3	230.2	<.001
Identifies as CALD (yes)	3.8	6.7	15.4	<.001
Identifies as indigenous (yes)	4.1	5.7	5.1	.023
Location (metropolitan)	69.6	66.5	4.2	.039
Ever lived together (yes)	94.0	95.7	5.8	.016
Communicated in the past 6 months (yes)	85.4	79.8	20.9	<.001
Children’s age profile			25.7	<.001
Young children only	30.5	46.2		
Older children only	57.2	49.1		
Both younger and older	12.4	14.4		
Spent time with younger children (yes)	85.3	91.6	18.3	<.001
Spent time with older children (yes)	85.0	90.4	18.6	<.001
	Mean (*SD*)	Mean (*SD*)	*t*	*P*
Age	38.5 (8.1)	37.3 (7.8)	4.70	<.001
Time since separation (years)	3.9 (3.1)	3.0 (3.0)	8.43	<.001

*Note*. CALD = culturally and linguistically diverse.

#### Patterns in Risk Profiles

Risks that tended to co-occur were sought using tetrachoric correlations, focusing on correlates of safety risks to oneself from the former partner, concerns for the safety of one’s own behavior/behaving in a risky manner toward the former partner and/or children, and concerns for the children’s safety because of the former partner’s behavior (see Supplemental Table 5 for the full correlation matrix). The highest correlate of risk to oneself was added safety fears associated with cultural or religious factors (*r*_tet_ = .51). The highest correlates of behaving in a risky manner were concerns about one’s own mental health and coping (*r*_tet_ = .62) and concerns for the children’s safety (*r*_tet_ = .62). Similarly, the strongest correlates of parental concerns for their children’s safety were concerns about their own mental health and coping (*r*_tet_ = .68) and concerns about their own behavior (*r*_tet_ = .62).

Factor analysis of the full tetrachoric correlation matrix identified four factors. Following rotation, Factor 1 (labeled “Difficulty parenting”) grouped together difficulty parenting (λ = .80) with younger children’s distress (λ = .69) and older children’s distress (λ = .78). Factor 2 (“Emotional distress”) comprised serious current conflict (λ = .88), negative emotions (λ = .79), and life stresses (λ = .50). Factor 3 (“Mental health”) grouped own mental health and coping (λ = .81) with the safety of one’s own behavior (λ = .72) and concern for the children’s safety (λ = .69). Factor 4 (“Safety fears”) paired fear associated with cultural or religious factors (λ = .62) and fears for one’s own safety (λ = .63). All other factor loadings were under 0.5 (see Supplemental Table 6 for the full rotated solution).

### Aim 2. Universal Utility and Fitness-for-Purpose of the DOORS Triage Tool

Given the Courts’ vested interest in ensuring that the risk screening and triage process is acceptable to vulnerable and marginalized groups, the submission time to engage with and submit the screen was examined among three key sub-groups of interest (high risk, Indigenous status, and CALD background). In keeping with Courts’ data that indicate over half of all parties submit the link within 48 hr of receiving the invitation, we found a modal time of 58 min (minimum 13 min), median time of 26 hr, and mean time 70 hr (2.5 days).

Predictors of short versus long submission time were assessed. Of the three key groups identified as being of concern, parties who identified as Aboriginal/Australian Indigenous and those who were classified as high risk on the DOORS Triage Tool were more likely to take a short time to engage with and submit the tool than their counterparts. Submission time did not vary by CALD background status. In addition, submission time was lower for applicants than respondents and for women than men. Older parties took longer to engage with and submit the tool than younger ones, but geographic location and children’s age profile were not associated with submission time (see Table 4).

**Table 4.** Parties’ Profiles by Submission Time (Short vs. Long) (% or Mean).

Variable	Short Time	Long Time	χ^2^	*P*
Applicant status	64.2	59.4	3.9	.049
Gender (male)	38.0	44.1	6.1	.013
Identifies as CALD (yes)	6.4	5.5	.7	.411
Indigenous (yes)	7.3	4.8	5.2	.022
Location (metropolitan)	68.0	67.7	.0	.881
Ever lived together (yes)	94.0	95.1	.0	.850
Communicated in past 6 months (yes)	83.2	81.8	.6	.447
Children’s age profile			2.0	.364
Young children only	36.9	33.7		
Older children only	50.6	52.4		
Both younger and older children	12.6	13.9		
Spent time with younger children (yes)	92.0	89.1	1.8	.185
Spent time with older children (yes)	89.2	88.1	.3	.598
Risk group (high)	67.1	61.2	6.0	.014
	Mean (*SD*)	Mean (*SD*)	*t*	*P*
Age	36.5 (7.9)	38.0 (7.9)	3.80	<.001
Time since separation (years)	3.4 (3.2)	3.4 (3.0)	0.03	.738

*Note*. CALD = culturally and linguistically diverse.

In subsequent multivariate logistic regression analyses controlling for gender, age, and applicant/respondent status, those designated as being at high risk continued to record shorter submission times than those at medium/low risk (Odds ratio 0.803, 95% CI [0.649, 0.994], *p* = .044). However, all other things being equal, Indigenous status was no longer associated with submission time (Odds ratio 0.714, [0.484, 1.05], *p* = .09).

### Aim 3. Accuracy of DOORS Triage Self-Reported Risk Against Actual Referral Pathways

Fitness-for-purpose of the DOORS Triage Tool was assessed by correspondence between the DOORS-based risk classification and later choice of referral pathway—to the high-risk Evatt List or standard pathway (i.e., the accuracy of the tool plus rubric). The referral is made by the Triage Counselor following further clinical assessment and review.

There was very high agreement between the DOORS-based risk classification and subsequent referral by Triage Counselors to be considered for the Evatt List (χ^2^ = 2,237.3, *p* < .001). A small proportion of respondents were classified as high risk but after assessment were later referred to the standard court pathway rather than the high-risk Evatt pathway (13.0%; “false positives”). Of those referred to the Evatt List, we identified individuals (1.2% of the sample) who were initially classified as low or medium risk on the DOORS Triage Tool but subsequently highlighted as higher risk (i.e., “false negatives,” Table 5). These cases were identified as a result of the Courts undertaking clinical review in response to other information or patterns of risk beyond the tool. Clinical review is not conducted for the majority of medium or low-risk matters.

**Table 5.** Comparison of DOORS Triage Tool Risk Classification and Later Referral to be Considered for the Evatt List (Cell Counts and Column Percentages).

Court-Verified Risk Level	DOORS Triage Tool Self-Reported Risk Classification							
	Self-Reported Low Risk		Self-Reported Medium Risk		Self-Reported High Risk		Total	
	*n*	%	*n*	%	*n*	%	*n*	%
Referred to Evatt List	42	4.7	7	1.0	2,037	79.0	2,086	50.0
Standard pathway	847	95.3	697	99.0	541	21.0	2,085	50.0
Total	889	100.0	704	100.0	2,578	100.0	4,171	100.0

The next set of analyses compared *confirmed referral to Evatt* (true high risk on DOORS to an Evatt referral) with *false positives* (high risk on DOORS to non-Evatt). This set of analyses highlighted groups of people whose risk status was later reclassified to a lower level. True positives numbered 2,037 and accounted for 48.8% of the total sample. False positives numbered 541 and accounted for 13.0% of the total sample. Hence, a classification of high-risk based on the DOORS Triage was accurate for nearly four times as many people as those for whom high risk on the DOORS was later reduced to medium or low risk.

Several sociodemographic, family-related, and risk variables were associated with reclassification (false positives) in bivariate analyses (see Supplemental Tables 7 and 8). When sociodemographic, family-related, and risk-related variables were combined in a backwards deletion multiple logistic regression analysis comparing true positives with false positives (*n* = 2,570), reclassification was associated with not reporting risk in three domains: 1 (fear for cultural/religious reasons), 9a (the party’s safety), and 9b (the party not behaving safely); and with male gender, non-Indigenous status, having communicated with the other party in the previous 6 months, longer time since separation, and not having a mix of older and younger children (see Supplemental Table 9).

## Discussion

This study reported on the utility of the DOORS Triage Tool (McIntosh et al., 2020) used within a family violence triage process in the Courts. Our findings offer three clear contributions to the literature that may inform policy and practice. Data from 4,175 DOORS Triage reports completed at or near entry to the Courts showed that:

1. *Self-reported family violence risk was consistent with subsequent independent assessment for 87% of parties*. In this light, the DOORS Triage Tool demonstrated utility and validity across the Courts’ population in differentiating high-risk cases from low-risk cases. That is, there was significant correspondence between DOORS-based high-risk classification and referral by Triage Counselors to the Evatt List following additional review of the case. In all, findings suggest the DOORS Triage Tool is a reliable and useful initial screen for high risk in the family courts.
2. *The most vulnerable sub-populations engaged with the screening opportunity*. Demographic factors associated with escalated risk included being a woman, being a respondent to the matter, and reporting Indigenous or CALD status. Those reporting high-risk and/or Indigenous status engaged most rapidly with the tool, suggesting satisfactory engagement with the triage process among the most vulnerable sub-groups.
3. *Patterns of risk within the tool are instructive for prevention, policy, and practice*. Our data highlighted critical links between ancillary safety risks for families, which may enhance early identification of and targeted response to families on entry to court. Our results suggest strong reciprocity between risk factors and imply the need to coordinate support across both risk and well-being domains of family life.

We consider these three areas in turn.

### Validity of Self-Reported Family Violence Risk

The validity of self-reported risk is routinely questioned, both in science and in the law. In science, especially concerning sensitive topics about mental health and safety, reasons for under-reporting risk on surveys include fear of being judged, endangered, or legally penalized (Blair et al., 2020). Furthermore, the structure of some self-report tools can create bias, for example, leading to greater gender parity in the reporting of domestic violence victimization than is suggested by established evidence about the predominance of women’s experience of partner abuse across the life course (Cunningham & Anderson, 2023; Hamby, 2016). For women in family court systems, particularly within CALD groups, the under-reporting of the severity or frequency of victimization experiences has long been understood as a response to fear of recrimination and escalated abuse (Morgan & Chadwick, 2009).

In these respects, two findings from this study are particularly important. First, DOORS Triage self-report corresponded highly with the confirmed risk classification through the Courts’ application of their risk rubric, and referral pathway, for men and women, across cultural groups. In other words, it provided a largely timely, efficient, and accurate self-report to commence the Courts’ triage process. Second, the screening process was acceptable for vulnerable sub-groups—namely, those who would later be identified as eligible for high-risk case management, and parents identifying as Indigenous.

Across the whole sample, self-reported risk and the resulting high, medium, or low classification aligned with the subsequent referral pathway in 87% of cases. The triage tool “missed” very few (1.2%) cases of those later reviewed in person and confirmed as high-risk. This result, together with high agreement rates between the high-risk self-report classification and subsequent referral by Triage Counselors to be considered for the Evatt List, can provide family courts with confidence that the triage tool offers an efficient, accurate beginning to risk identification and management.

Equally, there is something to learn from the proportion (21%) of respondents self-classified as high risk on the DOORS but whose risk was subsequently reclassified after Case File Review and, where offered, Triage Interview. Most in this group reported high risk to their children’s safety in the care of the other parent. We found a greater likelihood of exaggerated, false positive claims of risk among non-Indigenous men and those who had been separated for more than 2 years, were still in communication with their former partner, and had children aged over 6 years. Of interest in that group, few reported fears for their safety because of cultural/religious reasons, or concerns about the safety of their own behavior. The interactive risks of cultural diversity and victimization were clearly highest for women. This is consistent with the differential protective nature of culture and religious identification for men and women, reported elsewhere (Krahé, 2021).

### Gendered Risk Profiles

The DOORS Triage Tool includes two domains that ask directly about the safety of parent and child. The remaining domains tap into ancillary distress and well-being indices known to either amplify or ameliorate risks of being harmed, or risk of harming. Noting gender was self-identified, the DOORS Triage data showed far-ranging gendered differences in five out of the 10 domains. Our findings show a significant difference in the dominant empirical “narratives” evident on the tool for men and women, especially for those reporting high-risk. For women, the narrative is one tightly focused on fear for their safety. Women were significantly more likely than men to be the respondent in their proceedings, and to report risk to their own safety because of the behavior of their ex-partner and their cultural/religious background. For men, the pattern speaks of more wide-ranging distress. Men were significantly more likely than women to initiate court action, and to report strong negative emotion about being separated, concern about the former partner’s mental health and for the well-being and safety of their children, parenting difficulties, high life stress, and likelihood of perpetrating harm. The latter pattern of dysregulation and distress is one commonly reported as separation-related violence in the literature (Audet et al., 2022). Subsequent work by our team will explore finer distinctions by gender.

### Patterns of Risk Within the Tool Are Instructive for Prevention, Policy, and Practice

Our findings show there are critical links between ancillary risks for families, which can be reliably self-reported and may enhance rapid targeting of support to families on entry to court. In factor analysis, the first factor grouped parties’ worry about their children’s distress together with their own difficulty in parenting. Our results support the established science that children’s safety is inextricably connected to parenting practices, particularly sensitivity (Liel et al., 2022). Typically, a family court’s first response may not include the provision of parenting support, yet attention to parenting distress within the litigation process may be key to safety enhancement.

Secondly, we identified links between reports of serious ex-partner conflict with multiple ancillary life stresses, including isolation, employment, housing, and health. Most family court systems offer some form of parent education about conflict management, and calls are escalating for earlier and scalable offerings (O’Hara & Cohen, 2024). Our finding suggests the added need to address these reciprocal, reinforcing stressors in the socio-ecological context that amplify conflict and the impact of conflict on social functioning and financial hardship.

Thirdly, we identified associations between parents’ concerns about their own mental health with reports of their own unsafe behavior and with concern for the children’s safety. This finding in particular invites a systemic perspective on perpetration risk. Service capacity to detect those at risk of harming may be enhanced through concurrent self-reported mental health and social support concerns and concerns for their own children’s safety (Hunter et al., 2021). Attending to self-reported dysregulation across family and personal functioning domains appears key.

Finally, a fourth factor paired fears for one’s own safety with cultural or religious factors. Again, this key finding, replicated across analyses, highlights the need to enquire about cultural and religious affiliation and to note the potential amplification of risk status this may bring, especially for women (Olecká, 2022). Our findings on using the DOORS Triage Tool for early detection of risk in First Nations cultures are promising, as our results indicate the acceptability of this form of enquiry about family violence risk for Australian Indigenous parents.

### Strengths and Weaknesses

This research was conducted in a real-world setting, and its findings have direct implications for family courts. The data reported here have many strengths. The three pilot sites initially selected were chosen by the Courts to represent diverse parties and operationally different registries. Furthermore, the expansion of Lighthouse meant additional data comprising 32.5% of the sample were gathered from an additional 12 registries (except for Western Australia, which has its own state family law court and associated family law system).

The Courts’ annual published data during this time from the Notice of Child Abuse, Family Violence or Risk suggest the proportion of high-risk matters across the population is comparable to the proportion of high-risk classifications identified in our sample. However, given the voluntary nature of screening and limitations of consent, and the fact that low-risk cases were infrequently reviewed by a Triage Counselor, the data reported here clearly do not represent the entire court population during this period, and we may not have included important sub-groupings within high-risk categories. Such inclusions could have improved our estimates of the prevalence of risk factors and factors associated with high risk. Other weaknesses include our lack of details on how self-identity as CALD is related to risk, particularly for women, and as such in these areas, we cannot suggest implications for intervention.

### Conclusion

Confidential, voluntary, and universal family violence screening in the Courts is unique to the Australian Family Law context. Confidential risk triage processes are one way in which a busy court can discharge its obligations to those who require its services. Here, we have demonstrated the utility and validity of self-report within the Courts’ confidential triage processes, across demographic and socio-economic status groups. In this first examination of the implementation of a self-report “front end” to a triage process, the DOORS Triage Tool proved to be highly accessible and accurate across vulnerable sub-populations. Since the expansion of Lighthouse, 77% of eligible matters have at least one party completing the risk screen (Federal Circuit and Family Court of Australia, 2024). Our findings provide valuable information about the characteristics of family safety and risk on entry to the family court system, and a method that works well specifically in the context of self-reported risks being protected by legislation. As an important caveat, it would not be correct to apply our findings to family court systems where safety screening reports remain admissible as evidence.

Family law proceedings will always represent a period of escalating danger for many, especially for women. The DOORS Triage Tool and Courts’ risk rubric utilized at the front end of Lighthouse in the Courts shows strong potential to enhance risk identification and to notice clusters of risks that suggest enhanced case management. Our research is ongoing, exploring the economic and well-being benefits of systematic risk screening over time. In the interim, findings to date may reinforce for other family court systems the possibility and benefit of confidential risk screening and early triage.

## Supplemental Material

sj-docx-1-jiv-10.1177_08862605251363611 – Supplemental material for Family Violence Risk on Entry to the Family Courts of Australia: Profiles and Predictive Validity of the DOORS Triage Process Supplemental material, sj-docx-1-jiv-10.1177_08862605251363611 for Family Violence Risk on Entry to the Family Courts of Australia: Profiles and Predictive Validity of the DOORS Triage Process by Yvonne D. Wells, Jennifer E. McIntosh, Felicity L. Painter, Amy Holtzworth-Munroe, Bradley J. Wright, Heng Jiang, Jamie Lee, Kristel A. Krella, Anna T. Booth and Elizabeth Evans in Journal of Interpersonal Violence

## Data Availability

Given their sensitive nature, data for this study are subject to a data sharing agreement between the Federal Circuit and Family Court of Australia and La Trobe University and are not publicly available.
