# Supplementary material for: Family Violence Risk on Entry to the Family Courts of Australia: Profiles and Predictive Validity of the DOORS Triage Process
Source: J Interpers Violence. 2025 Aug 22;41(21-22):8375–401. doi: 10.1177/08862605251363611 (PMC13669445; doi:10.1177/08862605251363611)
Supplement: Supplementary file 1 [file sj-docx-1-jiv-10.1177_08862605251363611.docx]

**Supplementary Tables**

**Supplementary Table 1**

*Parties’ Sociodemographic Characteristics, Family Structure, and Overall Risk by Pilot vs. Expansion (% or Mean (SD))*

| Characteristic | Pilot  (n = 2817) | Post-pilot  (n = 1358) | χ^2^ | *p* |
| --- | --- | --- | --- | --- |
| Applicant status (Applicant) | 59.6 | 60.1 | 0.1 | .785 |
| Gender (Male) | 43.4 | 43.4 | 0.0 | .998 |
| Identifies as CALD (Yes) | 5.2 | 5.8 | 0.7 | .395 |
| Identifies as Indigenous (Yes) | 5.2 | 5.0 | 0.1 | .749 |
| Location (Metropolitan area) | 71.3 | 65.9 | 12.1 | < .001 |
| Ever lived together (Yes) | 93.2 | 96.0 | 15.4 | < .001 |
| Communicated in past 6 months (Yes) | 81.7 | 82.0 | 0.1 | .769 |
| Children’s age profile  Young children only  Older children only  Both younger and older children | 37.2  47.6  15.2 | 32.5  54.4  13.1 | 14.7 | < .001 |
| Spent time with younger children (Yes) | 88.9 | 89.7 | 0.4 | .552 |
| Spent time with older children (Yes) | 88.6 | 88.1 | 0.1 | .720 |
| High risk on the DOORS triage tool | 61.9 | 61.8 | 0.0 | .972 |
|  | Mean (SD) | Mean (SD) | *t* | *p* |
| Age | 36.8 (8.0) | 38.3 (7.7) | 5.73 | < .001 |
| Time since separation (yrs) | 3.3 (3.1) | 3.4 (3.0) | 1.04 | .299 |

**Supplementary Table 2**

*Sociodemographic Characteristics and Family Structure of Parties by Applicant/Respondent Status (% or Mean (SD))*

| Characteristic | Applicants  (n = 2502) | Respondents  (n = 1673) | χ^2^ | *p* |
| --- | --- | --- | --- | --- |
| Gender (Male) | 47.1 | 70.7 | 225.4 | < .001 |
| Identifies as CALD (Yes) | 5.6 | 5.6 | 0.0 | .953 |
| Identifies as Indigenous (Yes) | 5.4 | 4.6 | 1.2 | .280 |
| Location (Metropolitan) | 69.5 | 65.0 | 9.1 | .002 |
| Ever lived together (Yes) | 95.9 | 93.8 | 9.8 | .002 |
| Communicated in past 6 mo. (Yes) | 84.1 | 78.6 | 20.7 | < .001 |
| Children’s age profile  Young children only  Older children only  Both younger and older | 33.7  52.9  13.4 | 34.6  51.1  14.4 | 1.5 | .474 |
| Spent time with younger children (Yes) | 85.1 | 95.7 | 58.1 | < .001 |
| Spent time with older children (Yes) | 84.1 | 94.5 | 69.1 | < .001 |
|  | Mean (SD) | Mean (SD) | *t* | *p* |
| Age | 38.1 (8.0) | 37.4 (7.9) | 2.54 | .011 |
| Time since separation (yrs) | 3.4 (3.1) | 3.3 (2.9) | 1.9 | .057 |

**Supplementary Table 3**

*Risk Profile of Parties by Applicant/Respondent status (% high risk in each domain)*

| Variable | Applicant | Respondent | χ^2^ | *p* |
| --- | --- | --- | --- | --- |
| Domain 1 Fear re cultural/religious context | 7.8 | 8.8 | 1.4 | .244 |
| Domain 2 Negative emotions | 0.5 | 0.7 | 0.7 | .418 |
| Domain 3 Serious current conflict | 1.1 | 1.1 | 0.0 | .999 |
| Domain 4 Own mental health and coping | 6.6 | 7.5 | 1.3 | .251 |
| Domain 5 Other party’s mental health and coping | 14.4 | 14.2 | 0.0 | .879 |
| Domain 6a Young children’s distress | 4.1 | 1.3 | 12.6 | < .001 |
| Domain 6b Older children’s distress | 4.2 | 2.5 | 5.2 | .023 |
| Domain 7 Parenting difficulty | 2.1 | 1.1 | 5.0 | .026 |
| Domain 8 Concerns for children’s safety | 11.7 | 10.9 | 0.5 | .483 |
| Domain 9a Concerns for own safety | 53.8 | 66.0 | 61.6 | < .001 |
| Domain 9b Safety of own behaviour | 5.0 | 3.3 | 6.8 | .009 |
| Domain 10 Life stresses | 4.0 | 4.1 | 0.0 | .971 |

**Supplementary Table 4**

*Results of Logistic Regression of Predictors of High-Risk Status on the DOORS Triage Tool*

| Variable | *B* | S.E. | Wald | df | *p* | Odds ratio | 95% CI | |
| --- | --- | --- | --- | --- | --- | --- | --- | --- |
|  |  |  |  |  |  |  | Lower | Upper |
| Applicant/respondent | 0.28 | 0.07 | 14.6 | 1 | < .001 | 1.32 | 1.15 | 1.53 |
| Gender | 0.98 | 0.07 | 189.4 | 1 | < .001 | 2.66 | 2.31 | 3.05 |
| CALD background | 0.50 | 0.16 | 9.4 | 1 | .002 | 1.65 | 1.20 | 2.26 |
| Time since separation (years) | -0.11 | 0.01 | 87.1 | 1 | < .001 | 0.90 | 0.88 | 0.92 |
| Communicated in past 6 mo. | -0.34 | 0.10 | 13.0 | 1 | < .001 | 0.71 | 0.59 | 0.86 |
| Constant | -1.28 | 0.24 | 28.1 | 1 | < .001 | 1.32 | 1.15 | 1.53 |

**Supplementary Table 5**

*Correlation matrix: Tetrachoric correlations between risk on each domain (dichotomized to low/medium vs. high)*

| Domain | D1 | D2 | D3 | D4 | D5 | D6a | D6b | D7 | D8 | D9a | D9b | D10 |
| --- | --- | --- | --- | --- | --- | --- | --- | --- | --- | --- | --- | --- |
| D1 Fear re cultural/religious context | 1.00 | 0.14 | -0.01 | 0.26 | -0.06 | 0.08 | -0.11 | -0.05 | 0.24 | 0.51 | 0.20 | -0.08 |
| D2 Negative emotions | 0.14 | 1.00 | 0.77 | 0.51 | 0.30 | 0.59 | 0.51 | 0.60 | 0.37 | 0.21 | 0.32 | 0.63 |
| D3 Serious current conflict | -0.01 | 0.77 | 1.00 | 0.40 | 0.54 | 0.46 | 0.37 | 0.37 | 0.35 | 0.32 | 0.17 | 0.48 |
| D4 Own mental health and coping | 0.26 | 0.51 | 0.40 | 1.00 | 0.12 | 0.15 | 0.24 | 0.25 | 0.68 | 0.39 | 0.62 | 0.20 |
| D5 Other party’s mental health and coping | -0.06 | 0.30 | 0.54 | 0.12 | 1.00 | 0.43 | 0.40 | 0.40 | 0.26 | 0.08 | 0.17 | 0.36 |
| D6a Young children’s distress | 0.08 | 0.59 | 0.46 | 0.15 | 0.43 | 1.00 | 0.58 | 0.66 | 0.39 | 0.12 | 0.25 | 0.36 |
| D6b Older children’s distress | -0.11 | 0.51 | 0.37 | 0.24 | 0.40 | 0.58 | 1.00 | 0.76 | 0.52 | -0.01 | 0.41 | 0.49 |
| D7 Parenting difficulty | -0.05 | 0.60 | 0.37 | 0.25 | 0.40 | 0.66 | 0.76 | 1.00 | 0.43 | 0.10 | 0.33 | 0.57 |
| D8 Concerns for children’s safety | 0.24 | 0.37 | 0.35 | 0.68 | 0.26 | 0.39 | 0.52 | 0.43 | 1.00 | 0.44 | 0.62 | 0.25 |
| D9a Concerns for own safety | 0.51 | 0.21 | 0.32 | 0.39 | 0.08 | 0.12 | -0.01 | 0.10 | 0.44 | 1.00 | 0.37 | 0.04 |
| D9b Safety of own behaviour | 0.20 | 0.32 | 0.17 | 0.62 | 0.17 | 0.25 | 0.41 | 0.33 | 0.62 | 0.37 | 1.00 | 0.36 |
| D10 Life stresses | -0.08 | 0.63 | 0.48 | 0.20 | 0.36 | 0.36 | 0.49 | 0.57 | 0.25 | 0.04 | 0.36 | 1.00 |

**Supplementary Table 6**

*Results of factor analysis of tetrachoric correlation matrix, rotated solution (factor loadings ≥ .4)*

|  | Factor | | | |
| --- | --- | --- | --- | --- |
|  | 1 | 2 | 3 | 4 |
| Domain 7 Parenting difficulty | 0.80 |  |  |  |
| Domain 6b Older children’s distress | 0.78 |  |  |  |
| Domain 6a Young children’s distress | 0.69 |  |  |  |
| Domain 5 Other party’s mental health and coping | 0.44 | 0.41 |  |  |
| Domain 3 Serious current conflict |  | 0.89 |  |  |
| Domain 2 Negative emotions |  | 0.79 |  |  |
| Domain 10 Life stresses |  | 0.50 |  |  |
| Domain 4 Own mental health and coping |  |  | 0.81 |  |
| Domain 9b Safety of own behaviour |  |  | 0.72 |  |
| Domain 8 Concerns for children’s safety | 0.40 |  | 0.69 |  |
| Domain 9a Concerns for own safety |  |  |  | 0.64 |
| Domain 1 Fear re cultural/religious context |  |  |  | 0.62 |

**Supplementary Table 7**

*False Positives vs. True Positives by Sociodemographic and Family Characteristics (% or Mean)*

| Variable | True positives | False positives | χ^2^ | *p* |
| --- | --- | --- | --- | --- |
| Applicant Status | 54.4 | 58.6 | 3.0 | .084 |
| Gender (Male) | 31.1 | 46.4 | 44.2 | < .001 |
| Identifies as CALD (Yes) | 6.7 | 6.7 | 0.0 | .998 |
| Identifies as Indigenous (Yes) | 6.3 | 3.1 | 8.2 | .004 |
| Location (Metropolitan) | 66.5 | 66.4 | 0.0 | .959 |
| Ever lived together (Yes) | 96.2 | 93.9 | 5.3 | .021 |
| Communicated in past 6 mo. (Yes) | 77.7 | 87.8 | 27.2 | < .001 |
| Children’s age profile  Young children only  Older children only  Both younger and older | 36.3  48.0  15.8 | 36.2  53.0  10.7 | 9.6 | .008 |
| Spent time with younger children (Yes) | 91.2 | 92.9 | 0.8 | .385 |
| Spent time with older children (Yes) | 91.0 | 88.1 | 2.6 | .108 |
|  | Mean (SD) | Mean (SD) | *t* | *p* |
| Age | 37.1 (7.8) | 38.3 (18.3) | 3.32 | < .001 |
| Time since separation (yrs) | 2.9 (2.9) | 3.4 (3.1) | 2.81 | .005 |

**Supplementary Table 8**

*False Positives vs. True Positives by Risk Domain (%)*

| Variable | True positives | False positives | χ^2^ | *p* |
| --- | --- | --- | --- | --- |
| Domain 1 Fear re cultural/religious context | 14.5 | 8.3 | 14.4 | < .001 |
| Domain 2 Negative emotions | 0.8 | 1.3 | 1.3 | .262 |
| Domain 3 Serious current conflict | 1.7 | 1.9 | 0.1 | .784 |
| Domain 4 Own mental health and coping | 11.1 | 11.3 | 0.0 | .905 |
| Domain 5 Other party’s mental health and coping | 16.2 | 17.5 | 0.4 | .509 |
| Domain 6a Young children’s distress | 3.1 | 4.7 | 1.4 | .238 |
| Domain 6b Older children’s distress | 4.2 | 4.3 | 0.0 | .976 |
| Domain 7 Parenting difficulty | 2.1 | 2.1 | 0.0 | .896 |
| Domain 8 Concerns for children’s safety | 17.8 | 17.9 | 0.0 | .990 |
| Domain 9a Concerns for own safety | 95.3 | 92.1 | 9.1 | .003 |
| Domain 9b Safety of own behaviour | 7.5 | 4.8 | 4.9 | .027 |
| Domain 10 Life stresses | 4.8 | 5.0 | 0.1 | .820 |

**Supplementary Table 9**

*Results of Multiple Logistic Regression Analysis of Predictors of False versus True Positives*

| Variable | *B* | S.E. | Wald | df | Sig. | Odds ratio | 95% CI | |
| --- | --- | --- | --- | --- | --- | --- | --- | --- |
|  |  |  |  |  |  |  |  |  |
| Age | 0.01 | 0.01 | 2.8 | 1 | 0.090 | 1.01 | 1.00 | 1.03 |
| Gender | -0.66 | 0.10 | 37.7 | 1 | 0.000 | 0.52 | 0.42 | 0.64 |
| Indigenous status | -0.61 | 0.28 | 4.9 | 1 | 0.028 | 0.54 | 0.32 | 0.94 |
| In communication | 0.70 | 0.15 | 20.7 | 1 | 0.000 | 2.01 | 1.49 | 2.72 |
| Children’s age profile (ref. = young children only)  Older children only  Both older and younger children | -0.10  -0.43 | 0.14  0.17 | 6.3  0.5  6.2 | 2  1  1 | .042  .492  .013 | 0.91  0.65 | 0.69  0.46 | 1.19  0.91 |
| Time separated (years) | 0.05 | 0.02 | 6.8 | 1 | 0.009 | 1.05 | 1.01 | 1.09 |
| D1 Fear re cultural/religious context | -0.54 | 0.18 | 9.3 | 1 | 0.002 | 0.58 | 0.41 | 0.82 |
| D9a Concerns for own safety | -0.44 | 0.21 | 4.4 | 1 | 0.035 | 0.64 | 0.43 | 0.97 |
| D9b Safety of own behaviour | -0.83 | 0.24 | 12.0 | 1 | 0.001 | 0.44 | 0.27 | 0.70 |
| Constant | 0.97 | 0.66 | 2.2 | 1 | 0.139 | 2.64 |  |  |
